# Membrane binding of the insertion sequence of *Proteus vulgaris* L-amino acid deaminase stabilizes protein structure and increases catalytic activity

**DOI:** 10.1038/s41598-017-14238-7

**Published:** 2017-10-20

**Authors:** Yingchen Ju, Zhihong Liu, Zizhen Zhang, Lijun Duan, Qi Liu, Qiong Gu, Cheng Zhang, Jun Xu, Huihao Zhou

**Affiliations:** 1 Research Center for Drug Discovery, School of Pharmaceutical Sciences, Sun Yat-sen University, Guangzhou, 510006 China; 2 Department of Pharmacology and Chemical Biology, School of Medicine, University of Pittsburgh, Pittsburgh, 15261 USA

## Abstract

*Proteus vulgaris* L-amino acid deaminase (*pv*LAAD) belongs to a class of bacterial membrane-bound LAADs mainly express in genus *Proteus*, *Providencia* and *Morganella*. These LAADs employ a non-cleavable N-terminal twin-arginine translocation (Tat) peptide to transport across membrane and bind to bacterial surface. Recent studies revealed that a hydrophobic insertion sequence (INS) in these LAADs also interacts with bacterial membrane. However, the functional significance of INS-membrane interaction is not clear. In this study, we made site-directed mutagenesis on the surface-exposed hydrophobic residues of *pv*LAAD INS, and we found that these mutations impaired the INS-membrane interaction but did not affect *pv*LAAD activity in the solution. We further found that when cell membrane is present, the catalytic activity can be increased by 8~10 folds for wild-type but not INS-mutated *pv*LAAD, indicating that the INS-membrane interaction is necessary for increasing activity of *pv*LAAD. Molecular dynamic (MD) simulations suggested that INS is flexible in the solution, and its conformational dynamics could lead to substrate channel distortion. Circular dichroism (CD) spectroscopy experiments indicated that bacterial membrane was able to maintain the conformation of INS. Our study suggests the function of the membrane binding of INS is to stabilize *pv*LAAD structure and increase its catalytic activity.

## Introduction

L-amino acid oxidases/deaminases (LAAOs/LAADs, EC 1.4.3.2) are flavin-containing enzymes that catalyze the oxidative deamination of L-amino acids to corresponding α-keto acids with strict stereospecificity^1^. LAAOs/LAADs are widely expressed in snake venom, insects, mammals, fungi, fishes and some bacterial species^2–5^, and play various functional roles such as anti-microbes, anti-tumor, anti-leishmaniasis, anti-HIV, platelet aggregation inhibition and innate immune defenses of animals^6,7^. While most LAAOs/LAADs are secreted or cytosolic enzymes^8^, bacteria from genus *Proteus*, *Providencia and Morganella* express a class of membrane-bound LAADs^9^. Studies revealed that these membrane-bound LAADs could produce α-keto acids as siderophores to capture irons from the environment. *Proteus* is one of major pathogens causing infections in urinary tract (UTI) where the iron concentration is low^10^. Membrane-bound LAAD was suggested as an essential factor for *Proteus* survival since the knockout of LAAD was lethal for *Proteus mirabilis* ^11,12^. However, another study showed that the plasmid containing the LAAD gene was unable to restore the iron-limiting survival of a siderophore negative *E. coli* strain^13^, which put the physiological role of LAADs in debate. Despite this, these LAADs are gaining increasing interests for their potential applications on producing various α-keto acids in eco-friendly manners. Such as, phenylpyruvic acid^14^, α-Keto-γ-methylthiobutyric acid^15^, α-ketoglutaric acid^16^, α-keto isocaproate^17^ and pyruvate^18^ can be produced from their corresponding L-amino acids by either purified LAAD enzymes or the whole-cell biocatalysts overexpressing LAADs.

The bacterial membrane-bound LAADs encode a twin-arginine translocation (Tat) signal peptide at the N-termini, which exists in many extracellular flavin-containing proteins for their cross-membrane transportation^19^. Because of the sequence mutation at the cleavage site, the membrane-inserted signal peptide escapes from the peptidase cleavage after transportation and tethers LAADs onto the extracellular side of bacterial membrane^15,20^. When the transmembrane peptide was truncated, LAADs can still fold well in solution and present significant activity^16^. The activities can increase several folds when LAADs binding to bacterial membrane^20^. One possible reason is that the LAADs need other bacterial membrane proteins, such as cytochrome b, as the electron acceptor to re-oxidize the reduced cofactor FADH_2_ to FAD^21^. It is quite different from typical LAAOs/LAADs, which directly use O_2_ to re-oxidize the cofactor and generate H_2_O_2_ as the side product. Snake LAAOs were reported to inhibit bacterial growth by binding to bacterial surface and producing H_2_O_2_ locally^22,23^. The cofactor re-oxidization mechanism proposed for membrane-bound LAADs can also well explain why the LAADs do not inhibit the bacteria themselves. This proposed mechanism is supported by the following facts: 1) the H_2_O_2_ production has not been detected from *Proteus myxofaciens* LAAD (*pma*LAAD) even peroxidase/catalase inhibitor NaN_3_ was added, and 2) the O_2_ consumption and α-keto acid production were almost equal for *pma*LAAD even excess amount of catalase was added^24^. In contrast, because the catalase converts a H_2_O_2_ into one H_2_O and a half O_2_, the O_2_ consumption is half to the α-keto acid production in typical LAAOs/LAADs. Recently, purified *pv*LAAD was found to produce significant amount of H_2_O_2_ *in vitro* without bacterial membrane, suggesting LAADs could alternatively utilize O_2_, although less efficient, to re-oxidize FADH_2_^25^.

The crystal structures of bacterial membrane-bound LAADs from *Proteus myxofaciens* (*pma*LAAD) and *Proteus vulgaris* (*pv*LAAD) have been reported^21,25^. This opens a door to understand the catalytic mechanism of this class of enzymes and invent the improved biocatalysts^26^. A hydrophobic insertion sequence (INS) was discovered in both *pv*LAAD and *pma*LAAD^21,25^. The deletion of INS in *pv*LAAD can have *pv*LAAD almost completely lose the catalytic activity. This implies that the INS plays an important role in catalytic process. The INS of *pv*LAAD (from Val321 to Met375) is located nearby the active site, and forms a hydrophobic module on protein surface. Structural analysis and liposome-binding assays suggested that the INS interacts with bacterial membrane^25^. However, it is not known whether membrane binding of INS plays a role in catalysis.

In the present study, we found that INS-mediated membrane-binding is responsible for membrane-caused catalytic activity enhancement and it also significantly changes the substrate preference. Our molecular dynamic (MD) simulations found that the INS is flexible before binding to membrane and tended to disturb the conformation of the substrate tunnel and catalytic center. Circular dichroism difference spectroscopy (CD) results indicate that the INS-membrane interaction stabilizes the INS structure. We propose that the structure stabilization is a mechanism to enhance the activity for bacterial membrane-bound LAADs.

## Results

### Binding with the lipid bilayer enhances the catalytic activity of *pv*LAAD

Membrane electron transfer chain-mediated cofactor re-oxidization is an important mechanism for bacterial membrane to enhance LAADs activities. To figure out whether it is the sole mechanism for the activity enhancement by membrane, we detected the catalytic activity of *pv*LAAD associated with the membrane whose electron transfer chain was blocked or not.

**Figure 1:**
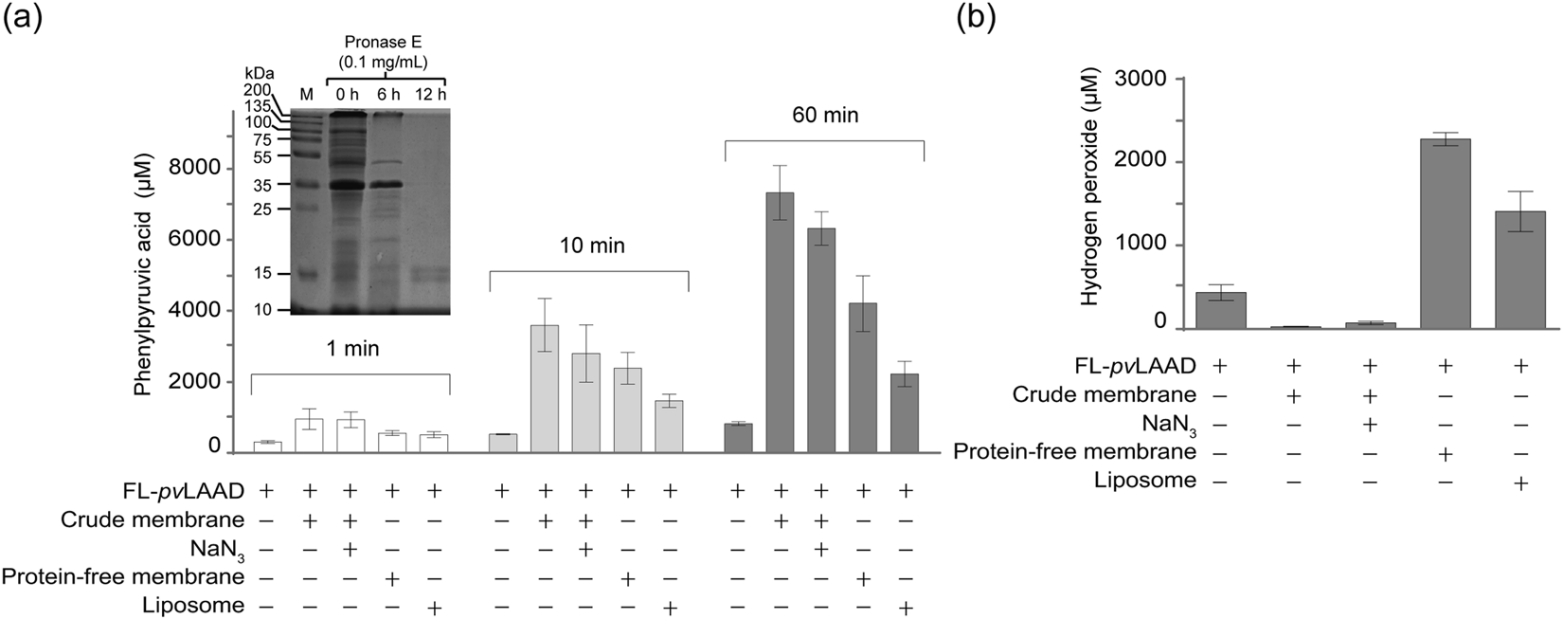
Membrane binding enhances *pv*LAAD activity. (**a**) Catalytic activities of *pv*LAAD with or without membrane presence were tested by measuring the transformation of L-phenylalaine to phenylpyruvic acids (PPA) in 1, 10 and 60 min. The activity of full-length *pv*LAAD with *E. coli* crude membrane was significantly enhanced compared to that without membrane, and the enhancement was only slightly reduced by adding the cytochrome oxidase inhibitor sodium azide. Pronase E-treated protein-free membrane, as well as the liposome, also holds the capability to enhance the enzymatic activity, although the fold change is smaller than crude membrane. The results here and later are all from three independent assays, and the error bars are SEM (standard error of the mean). The inset shows that almost all of the proteins on the *E. coli* crude membrane have been digested after 12 hr of treatment with 0.1 mg/mL pronase E at 37 °C. (**b**) The production of H_2_O_2_ was measured by HRP-coupled methods. Significant enhancement of H_2_O_2_ production in the reactions containing protein-free membrane and the liposome, indicating the O_2_ was used as the substrate by *pv*LAAD in the deamination of amino acids. H_2_O_2_ production has not been detected in the reactions with the crude membrane, which might be due to the strong catalase activity associated with crude membrane.

When membrane was added (2 mg/mL), 10 μM of purified full-length *pv*LAAD (FL-*pv*LAAD) produced 7655 ± 320 μM of PPA in 60 min, which was 9.3-fold higher than that of *pv*LAAD without membrane (820 ± 35 μM). Further increase of *pv*LAAD activity by adding more excess bacterial membrane was not significant (Supplementary Fig. S1). When 10 mM of sodium azide, an inhibitor of cytochrome oxidase of the electron transfer chain^27^, was added, the activity of *pv*LAAD with membrane was only slightly reduced (6382 ± 236 μM of PPA in 60 min), and still remained 7.8-fold higher than that of *pv*LAAD without membrane (Fig. 1a). This suggested that a mechanism other than the electron transfer chain-coupling is involved in membrane-caused activity enhancement of *pv*LAAD.

The crude extract of *E. coli* membrane contains a large amount of membrane integrating and association proteins, which may play roles in membrane-associated activity enhancement of *pv*LAAD. Pronase E was employed to treat the membrane. Protein electrophoresis showed that almost all of the membrane proteins have been digested after 12 h treatment with 0.1 mg/ml pronase E at 37 °C (the insert of Fig. 1a). This protein-free *E. coli* membrane still significantly increased the activity of *pv*LAAD (5.2 folds at 60 min). This result suggested that the membrane-caused activity enhancement did not completely rely on the functional membrane proteins. To further test this idea, liposomes without any functional proteins were constructed using *E. coli* lipids and incubated with *pv*LAADs. The PPA production was still increased to 2.9 folds (in 60 minutes). The smaller fold change by liposomes than *E. coli* membrane can be due to the incomplete reconstruction of liposomes from lipid molecules.

At the same time, the production of H_2_O_2_ was also quantified. Our results showed that the H_2_O_2_ production was significantly increased by adding protein-free *E. coli* membrane or liposomes, and the fold change was similar to that of the α-keto acid production (Fig. 1b). No H_2_O_2_ was observed when *pv*LAAD was incubated with crude membrane, which was due to the strong catalase activity associated with *E.coli* membrane^28^. In conclusion, our results suggested that both cell membrane and artificial lipid bilayer can significantly enhance the catalytic activity of *pv*LAAD, and O_2_ can be used to re-oxidize the cofactor FAD if the membrane electron transfer chain was blocked.

### INS-membrane interaction is important for activity enhancement of *pv*LAAD

*pv*LAAD has two membrane-binding elements. The N-terminal transmembrane Tat peptide commonly exists in many membrane-bound or secreted proteins containing redox cofactors^19,29^. Its function is more likely for providing membrane binding affinity rather than catalytic activity regulation. Consistently, truncating the N-terminal Tat peptide (ΔN-*pv*LAAD, res.30–471) strongly reduces membrane binding of *pv*LAAD^25,30^, but does not affect the catalytic activity of *pv*LAAD in solution (Supplementary Fig. S2). The INS is an assistant membrane-binding element identified in membrane-bound LAADs^25^. It locates near the catalytic site and is involved in forming the substrate channel. *pv*LAAD without INS ((res.1–325)-GGSS-(res.375–471), ΔINS-*pv*LAAD) lost all of the catalytic activity, suggesting that the INS plays an critical role in the catalytic activity(Supplementary Fig. S2).

**Figure 2:**
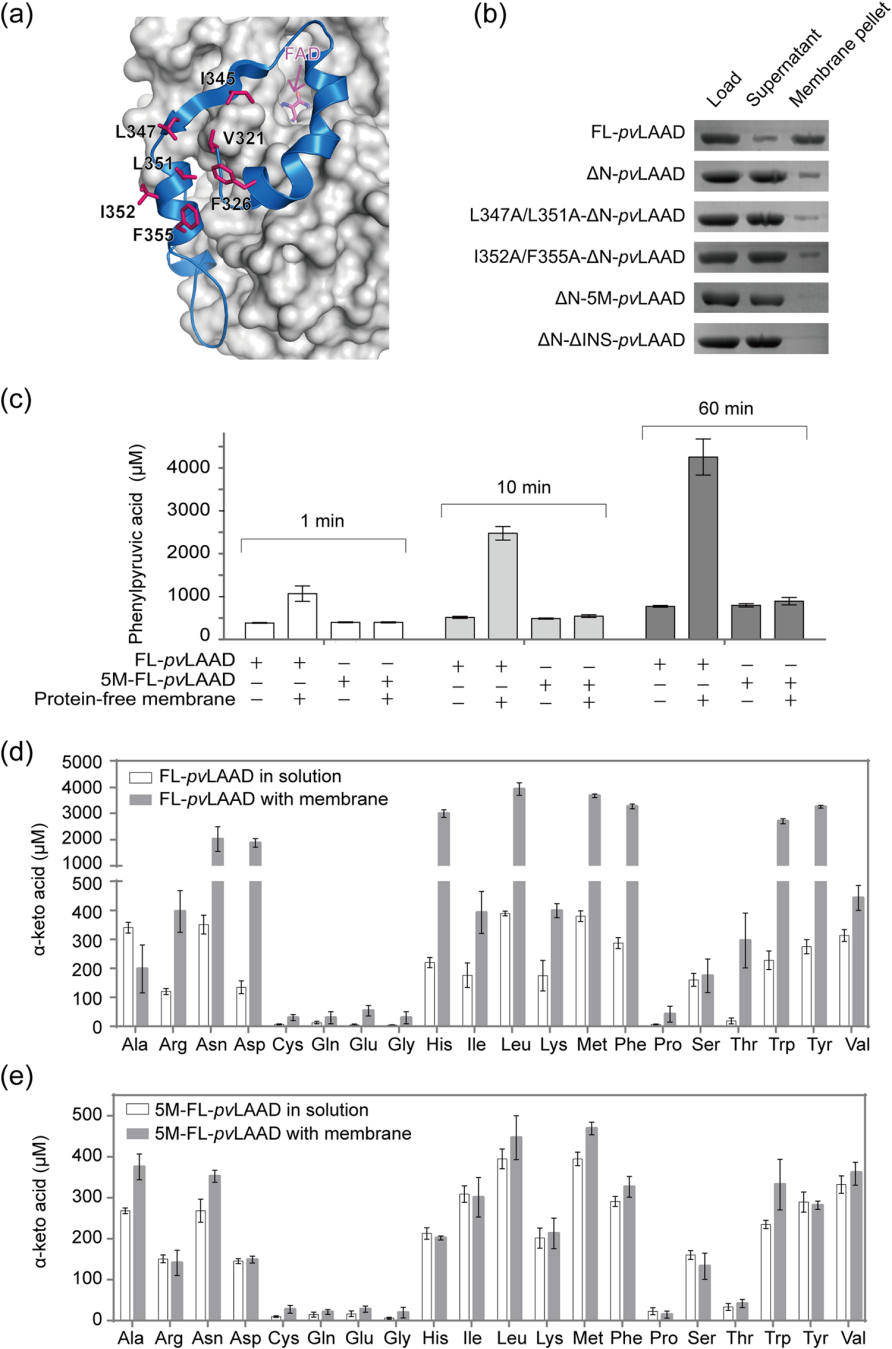
The INS-membrane interactions enhance the enzymatic activities and modulate the substrate preference of *pv*LAAD. (**a**) The cartoon drawing of the INS (blue) of *pv*LAAD. Seven of the conserved surface-exposed hydrophobic residues are drawn as sticks (magenta). (**b**) Membrane binding of INS-mutated *pv*LAADs were detected by membrane co-pelleting assays. *pv*LAADs co-pelleting with membrane or staying in supernatant were analyzed with SDS-PAGE. FL-*pv*LAAD was used as the positive control, and the double-truncated ΔN-ΔINS-*pv*LAAD was used as the negative control. Although the N-terminal Tat peptide is the major membrane-binding site of *pv*LAAD, a small fraction of ΔN-*pv*LAAD still co-pelleted with the liposome through the INS-mediated membrane binding. Two double-mutated ΔN-*pv*LAAD proteins showed the similar membrane-binding capability to the wild-type ΔN-*pv*LAAD. But, co-pelleting of the quintuple-mutant ΔN-*pv*LAAD (5M-ΔN-*pv*LAAD) could not be observed, and all of the 5M-ΔN-*pv*LAAD protein stayed in the supernatant, indicating that the quintuple mutation completely abolished the membrane-binding capability of the INS. (**c**) Catalytic activities of FL-*pv*LAAD and 5M-FL-*pv*LAAD with or without membrane presence were tested at three different time points (1, 10 and 60 min). FL-*pv*LAAD and 5M-FL-*pv*LAAD gave the similar activity when in the solution, but activity enhancement by membrane binding could be observed only for wild-type FL-*pv*LAAD but not for 5M-FL-*pv*LAAD. (**d**,**e**) Catalytic activities of wild-type FL-*pv*LAAD and 5M-FL-*pv*LAAD against twenty proteinogenic L-amino acids were tested by measuring the α-keto acid production using chromogenic reaction with 2,4-dinitrophenylhydrazine (DNP). Membrane binding enhanced the activities of FL-*pv*LAAD for most of the twenty proteinogenic L-amino acids, but with different folds. The highest activities were observed for Asn, Asp, His, Leu, Met, Phe, Trp and Tyr when membrane was added, which are significantly different to the favorable substrates of *pv*LAAD without membrane. In contrast, no activity enhancement and substrate spectrum shift were observed for the 5M-FL-*pv*LAAD.

Previous structural analysis suggested that the INS binds to membrane through hydrophobic interactions^25^. The INS contains several surface-exposed hydrophobic residues, and seven of them (V321, F326, I345, L347, L351, I352 and F355, Fig. 2a) are conserved in LAADs among genera *Proteus* and *Providencia* bacteria (Supplementary Fig. S3)^25^. Mutating these residues were expected to block the hydrophobic interactions between the INS and membrane. Double mutations (L347A/L351A-ΔN-*pv*LAAD, I352A/F355A-ΔN-*pv*LAAD) and quintuple mutations (I345A, L347A, L351A, I352A and F355A, named as 5M-ΔN-*pv*LAAD) were generated (Supplementary Fig. S3), and their binding with membrane were tested. FL-*pv*LAAD was used as the positive control, and double-truncated ΔN-ΔINS-*pv*LAAD was used as a negative control. Because the N-terminal Tat peptide is the major membrane-binding site^25^, most ΔN-*pv*LAAD stayed in the supernatant and only a small amount of ΔN-*pv*LAAD co-pelleted with the *E. coli* membrane (Fig. 2b). Double mutations (L347A/L351A-ΔN-*pv*LAAD, I352A/F355A-ΔN-*pv*LAAD) did not further reduce the membrane binding of ΔN-*pv*LAAD. However, the mutations (5M-ΔN-*pv*LAAD) completely abolished the membrane binding (Fig. 2b). The catalytic activity of *pv*LAAD mutants in solution was also measured. All the *pv*LAAD mutants, except ΔN-ΔINS-*pv*LAAD, kept the similar catalytic activity compared to the wild-type FL-*pv*LAAD (Supplementary Fig. S4), suggesting that the quintuple mutations only blocked INS-membrane interaction but did not affect the catalytic activity of *pv*LAAD in solution.

The N-terminal Tat peptide was added back to 5M-ΔN-*pv*LAAD to generate 5M-FL-*pv*LAAD, which could anchor to membrane through the N-terminal peptide. Without membrane, FL-*pv*LAAD and 5M-FL-*pv*LAAD exhibits the similar activity in solution. When *E. coli* protein-free membrane was added (2 mg/mL), the PPA production by FL-*pv*LAAD reached to 4325 ± 290 μM in 60 min, which was about 5.5-fold higher than that by FL-*pv*LAAD without membrane (780 ± 35 μM in 60 min). In contrast, no obvious activity increase were observed for 5M-FL-*pv*LAAD when *E. coli* membrane was added (803 ± 52 μM versus 962 ± 75 μM in 60 min), and the PPA production by 5M-FL-*pv*LAAD was similar to wild-type FL-*pv*LAAD in solution (Fig. 2c). The amount of H_2_O_2_ produced by *pv*LAADs were also detected after 60 min. When membrane was added, the significant increase of H_2_O_2_ production was only observed for wild-type FL-*pv*LAAD (about 4.7 folds) but not for 5M-FL-*pv*LAAD (Supplementary Fig. S5), which is similar to the situation of PPA production. These results indicate that the INS-mediated membrane-binding plays a major role in enhancing the catalytic activity of *pv*LAAD.

### Membrane binding regulates *pv*LAAD substrate spectrum

To prove the membrane binding can affect the substrate preference of *pv*LAAD, all the 20 proteinogenic L-amino acids were tested with or without adding membrane for wild-type FL-*pv*LAAD and 5M-FL-*pv*LAAD. In solution, two proteins showed the same substrate preference that L-alanine, L-asparagine, L-leucine, L-methionine, L-phenylalanine, L-tryptophan, L-tyrosine and L-valine are the preferred substrates for both proteins (Fig. 2d and e). When bacterial membrane was added, the activities of FL-*pv*LAAD against L-asparagine, L-aspartate, L-histidine, L-leucine, L-methionine, L-phenylalanine, L-threonine, L-tryptophon and L-tyrosine were increased by 6.8–13.6 folds, and the activities against the L-arginine, L-isoleucine, L-lysine were increased by 1.6–3.3 folds. In contrast, no significant activity increase was observed against L-alanine, L-serine and L-valine (Fig. 2d). Other L-amino acids, such as L-cysteine, L-glutamate, L-glutamine, L-proline and glycine, are not preferred substrates of *pv*LAAD, although some activity enhancement by membrane binding could also been observed for these amino acids. Because of the different fold changes, the top substrates of FL-*pv*LAAD switched to L-asparagine, L-aspartate, L-histidine, L-leucine, L-methionine, L-phenylalanine, L-tryptophon and L-tyrosine when membrane was present, and L-alanine and L-valine were no longer the preferred substrates.

The substrate preference of 5M-FL-*pv*LAAD is similar to FL-*pv*LAAD in solution. They have the same activity for most of the twenty L-amino acids except L-isoleucine, which is more preferred by 5M-FL-*pv*LAAD (Fig. 2e). Although 5M-FL-*pv*LAAD can still bind to membrane through the N-terminal Tat peptide, however, Tat peptide-mediated membrane binding did not cause significant activity change to any of the twenty L-amino acids (Fig. 2e). Therefore, membrane did not affect the substrate preference for 5M-FL-*pv*LAAD. These results indicated that only the INS-mediated membrane binding could modulate the substrate preference of *pv*LAAD.

### Structural flexibility of the INS

**Figure 3:**
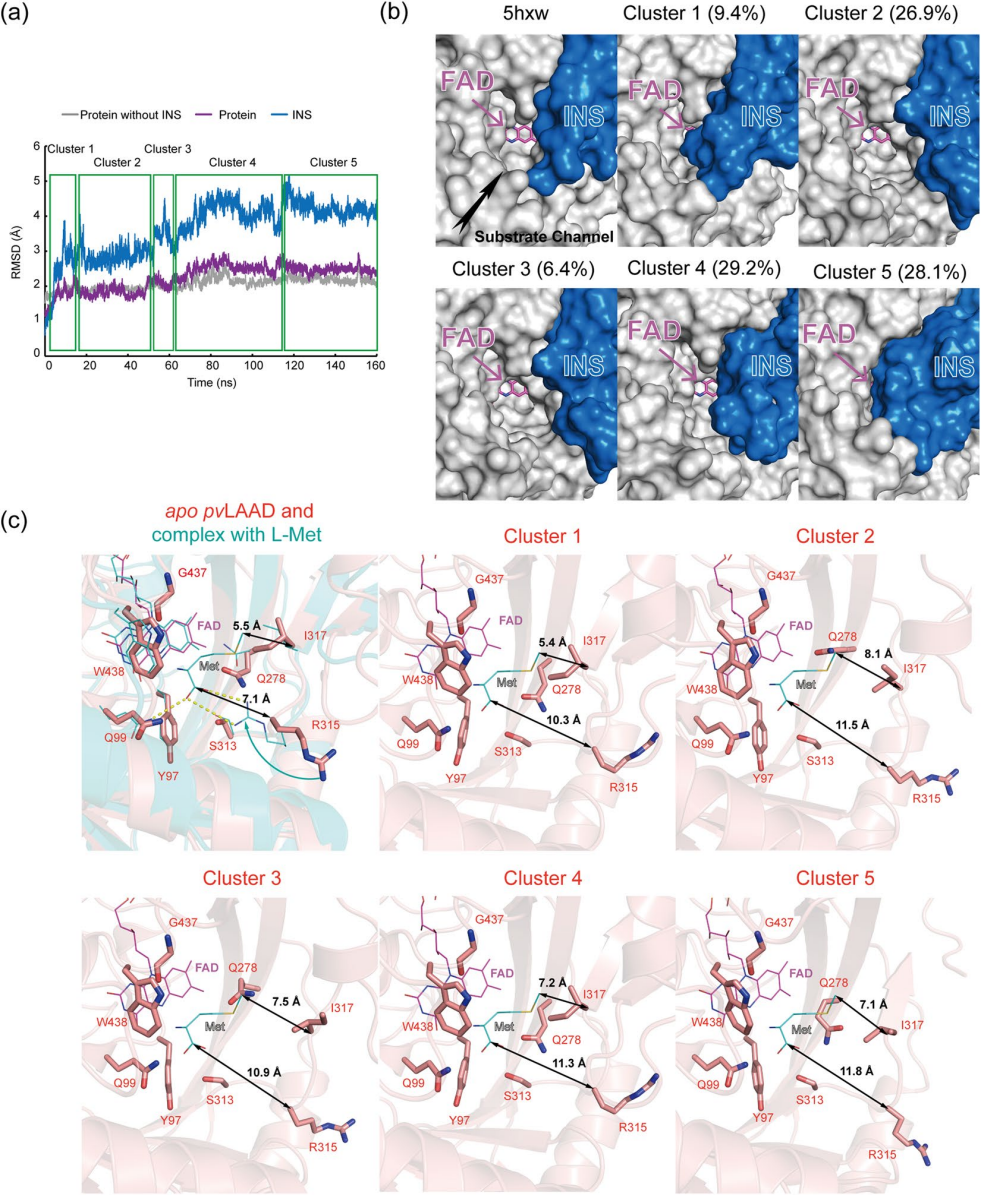
Molecular dynamics simulations of *pv*LAAD. 160 ns of MD simulation was performed with AMBER12 program package using the crystal structure of ΔN-*pv*LAAD (PDB code 5hxw) as the start model. (**a**) The RMSD-based trajectory analysis showed that the overall structure of ΔN-*pv*LAAD (purple) was stable, but the INS was flexible. The conformations of the INS were clustered to five classes. (**b**) The representative structures of each cluster. The INS was colored blue and the rest part of *pv*LAAD was colored white. The INS showed large movement among different clusters. The substrate channel opened in cluster 2 (26.9% population) and cluster 3 (6.4% population), but tended to close in cluster 1, 4 and 5. (**c**) The catalytic pockets of the representative structures of each cluster. While key residues Y97, Q99, Q278, S313, G437 and W438 were stable during MD simulations, residue R315, which could interact with the carboxyl group of amino acid substrates, and residue I317, which could contact with the side chain of the substrate amino acids, deviate from their initial positions in catalytic site during MD simulations. The *apo pv*LAAD structure (PDB code 5hxw) was colored orange, and its complex with L-methionine was modeled^24^ and colored cyan.

The crystal structures of *pv*LAAD and *pma*LAAD have been determined^21,25^. Their overall structures are quite similar to each other. The root-mean-square derivation (RMSD) between two structures is 0.723 Å for 386 comparable Cα atoms. The INS in both structures consists of three α-helices and one β-strand, and their structural differences are mainly in the first α-helix and the following loop links to the β-strand, which are closed to the substrate channel. The B-factors of the residues in the first α-helix and the following loop are very high in both structures, implying a dynamic structure. MD simulations were conducted to study the dynamic conformations of the INS using the crystal structure of ΔN-*pv*LAAD (PDB code 5hxw) as the start conformation. The RMSD of the systems is reasonable and achieves equilibrium during the simulation, indicating that the force field and simulation protocol used here are adequate for the current systems (Fig. 3a). The structure of *pv*LAAD excluding the INS stayed stable during simulation. In contrast, the INS underwent dramatic conformational rearrangements, indicating the highly dynamic nature of the INS in the solution phase. The confirmations of the INS were clustered, and the representative structures for each cluster were depicted in Fig. 3b. In the INS, the first and second α-helices and the short β-strand were more dynamic compared to the third α-helix in all clusters. They showed large movement during simulations, and their secondary structure was destructed in some clusters. Because of their movement, the substrate channel was closed in cluster 1 (9.4% population), cluster 4 (29.2% population) and cluster 5 (28.1%), and was opened only in cluster 2 (26.9% population) and cluster 3 (6.4% population) (Fig. 3b).

At the catalytic center, most residues (such as Tyr97, Gln99, Gln278, Ser313 and Gly437) were stable during MD simulations (Fig. 3c). However, residues Arg315 and Ile317 are just a few residues before INS on the protein sequence, and the conformation rearrangement of the INS moved both residues a few angstroms away from their starting positions during the simulations (Fig. 3c). Arg315 is the key residue for stabilizing the carboxyl group of substrate amino acids shown by previous MD simulations of *pv*LAAD complexed with L-methionine and by enzyme-inhibitor cocrystal structure of *pma*LAAD (Arg316 in *pma*LAAD)^21^. The mutation of this residue could significantly reduce the catalytic activity of *pv*LAAD^25^. Ile317 is the key residue that interacts with the side chain of the substrate L-amino acids, and mutation of this residue changed the substrate spectrum of *pv*LAAD^25^. The structural instability of these key residues in catalytic center would reduce the substrate binding affinity and slow down the transformation rate of *pv*LAAD in the solution.

### Membrane binding stabilizes the INS structure

**Figure 4:**
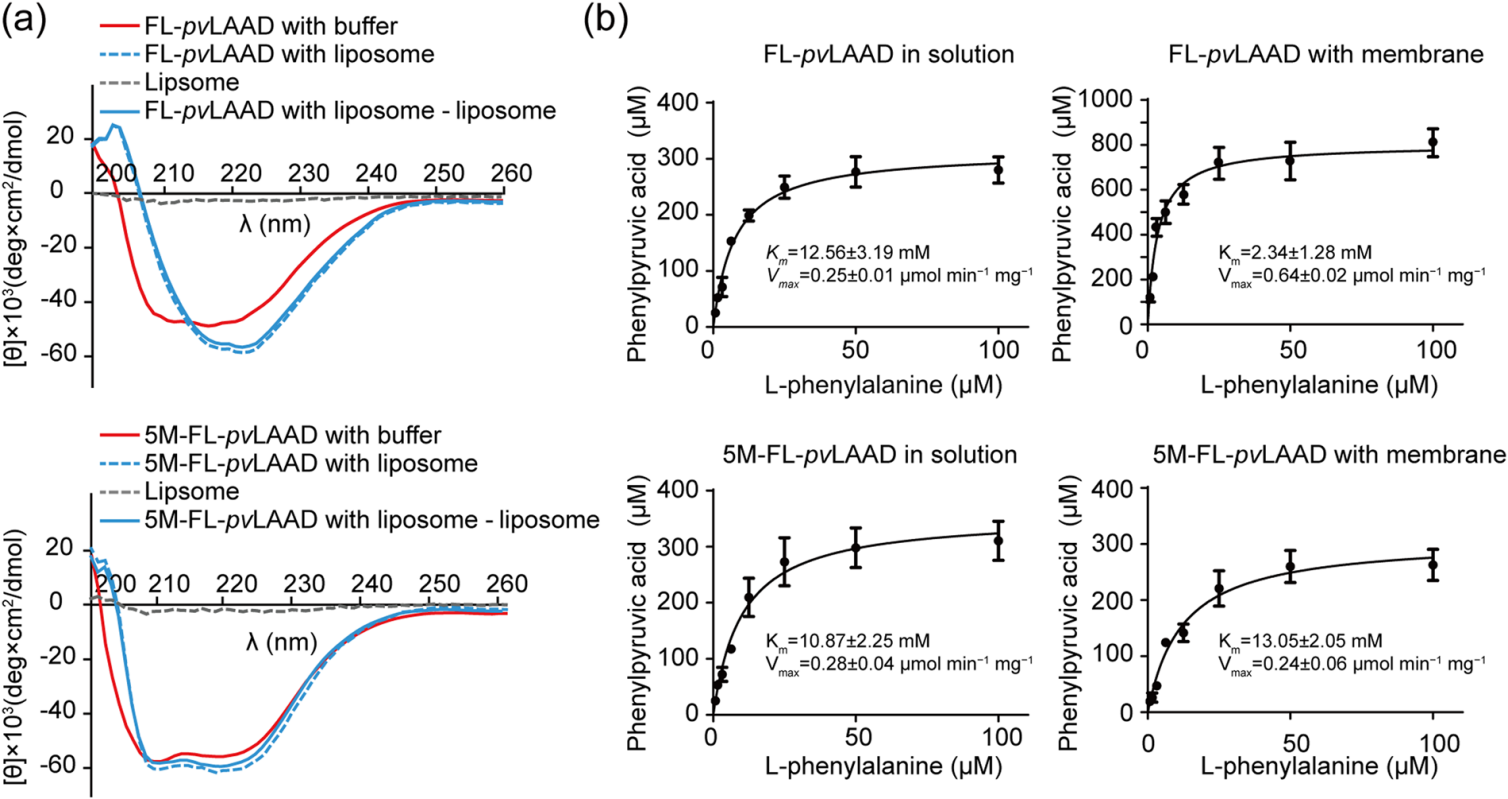
Membrane binding stabilized the structure of *pv*LAAD. (**a**) Liposome-binding-caused secondary structure changes of the wild-type and mutated *pv*LAAD were detected by CD. The CD spectra of FL-*pv*LAAD and 5M-FL-*pv*LAAD were recorded with or without adding the liposome. The CD spectrum of the liposome was also recorded (dashed line), and was subtracted as background from the spectra of *pv*LAADs with the liposome. An obvious bathochromic shift was observed between the CD spectra in solution (Red line) and with liposome (Blue line) of FL-*pv*LAAD. However, such bathochromic shift was less significant for 5M-FL-*pv*LAAD, suggesting the secondary structure reorganization was mainly caused by the INS-liposome interaction. (**b**) Enzymatic kinetic parameters of FL-*pv*LAAD and 5M-FL-*pv*LAAD with or without membrane presence were measured. In comparison with *pv*LAAD in the solution, membrane enhanced the substrate binding affinity (*K*_*m*_) for about 5.4 folds and substrate transformation rate (*V*_*max*_) for about 2.6 folds for wild-type *pv*LAAD. The enzymatic kinetic parameters of 5M-FL-*pv*LAAD were quite similar to those of wild-type *pv*LAAD in the solution, and they were not changed by adding the membrane.

The structural dynamics of the INS might impair the catalytic activity of *pv*LAAD in the solution, therefore, we wonder if the INS-membrane interaction can stabilize the INS structure. The circular dichroism (CD) spectra of FL-*pv*LAAD and 5M-FL-*pv*LAAD were recorded with or without liposome presence. The spectrum of liposome alone was also recorded and subtracted in calculation. The CD spectra of FM-FL-*pv*LAAD with and without liposome presence were similar and only a small shift was observed (Fig. 4a), indicating that the liposome binding of N-terminal Tat peptide only caused a small structural change. In contrast, a significant shift was observed between the two CD spectra of the wild-type FL-*pv*LAAD, suggesting that the INS-liposome interaction caused a significant secondary structure reorganization (Fig. 4a). The percentages of secondary structure components of FL-*pv*LAAD were calculated based on the CD spectra using K2D method^31^. The calculated secondary structure components of *pv*LAAD based on CD spectra were closed to those shown in the crystal structures (Supplementary Table S1). The results of FL-*pv*LAAD with liposome presence revealed that the random coil content decreased by 5% (from 45% to 40%), the β-sheet content increased by 2% (from 24% to 26%) and the α-helix content increased by 3% (from 31% to 34%). The increase of the secondary structure content indicated that the INS-membrane interaction stabilizes the structure of the INS.

Then, we studied how the structural stabilization effect by INS-membrane interaction could improve the catalytic kinetics of FL-*pv*LAAD. The catalytic activities of FL-*pv*LAAD and 5M-FL-*pv*LAAD were measured with or without membrane (Fig. 4b). When a wild-type FL-*pv*LAAD was incubated with the membrane, the substrate affinity increased by about 5.3 folds (Km value was changed from 12.56 ± 3.19 mM to 2.34 ± 1.28 mM), and the maximal velocity of the reaction increased by about 2.6 folds (*V*_*max*_ was changed from 0.25 ± 0.01 μmol min^−1^ mg^−1^ to 0.64 ± 0.02 μmol min^−1^ mg^−1^). In contrast, the membrane did not affect the activity of 5M-FL-*pv*LAAD. With or without membrane presence, the enzymatic kinetic parameters of mutated *pv*LAAD were similar to those of wild-type *pv*LAAD without membrane (Fig. 4b). These results proved that the membrane binding-mediated structural stabilization of the INS can enhance both the substrate binding affinity and transformation rate, while substrate binding was significantly enhanced.

## Discussion

The LAADs from genera *Proteus*, *Providencia* and *Morganella* bacteria are different from the typical LAAOs/LAADs in two aspects: (1) these bacterial LAADs are membrane-bound enzymes and their activities are several folds higher when on membrane than in the solution; (2) these LAADs do not produce H_2_O_2_ during catalytic process. It was believed that the bacterial LAADs cannot efficiently transfer the electrons directly to O_2_ for cofactor re-oxidization. Instead, they transfer electrons to membrane electron transfer chain. This hypothesis could well explain why the membrane could enhance the activity of these bacterial LAADs, and also explain why these LAADs do not generate H_2_O_2_, a strong oxidant that may cause severe damage to bacterial membrane. However, this hypothesis could not explain the fact that the protein-free membrane could still significantly enhance the activity of LAADs *in vitro*. *pv*LAAD has two membrane binding sites. The N-terminal non-cleavable Tat signal peptide was the major membrane-binding site, but we found Tat peptide-mediated membrane binding could not increase the enzymatic activity. Because the central helix of Tat peptide is less hydrophobic, its binding to membrane is weaker than typical transmembrane helices^32^. We previously proposed that the INS of bacterial LAADs may serve as an assistant membrane-binding site to avoid the risk of dissociating from the cell membrane^25^.

In the current study, we found that the membrane-mediated structure stabilization is a new mechanism, which is independent to the mechanism of the electron transfer chain-coupled cofactor re-oxidization, for how the membrane binding enhance the catalytic activity of *pv*LAAD. Membrane binding-induced structure stabilization is involved in the enzymatic activation in many peripheral membrane-bound proteins, such as RNase E^33^. RNase E contains a conserved segment, which has the propensity to form an amphipathic α-helix to mediate membrane binding. This membrane binding stabilizes RNase E in its activate conformation, which leads to a stronger substrate binding affinity^34^. Another case is the *E. coli* pyruvate oxidase (*Ec*POX). When *Ec*POX stays in the cytosol, its C-terminal domain covers the active site and blocks the access of the substrate^35^. The membrane binding of the C-terminus stabilizes *Ec*POX at an open conformation, which makes its active site fully accessible to the substrate pyruvate and electron acceptor Q8 and increases the catalytic activity for about 30 folds^35^. *pv*LAAD and other membrane-bound LAADs are the new examples of membrane binding-induced enzyme activation. We propose that protein engineering which stabilize the INS-membrane interactions could help to maintain the active conformation of LAADs, therefore results in the biocatalyst with higher transformation activity.

The membrane-binding affinity of the INS is relatively weak. It is possible that the INS can switch between the membrane-association and membrane-disassociation states. This may make the enzyme switch between the fully active and partially active states. Based on the current data of bacterial membrane-bound LAADs, we suggest that the two membrane-binding elements have evolved two different roles. The N-terminal Tat peptide is responsible for the cross-membrane transportation of the LAAD and finally anchoring it to out-surface of bacterial membrane where the enzyme activity is required. The INS is in charge of the enzyme activation when the enzyme activity is needed (Supplementary Fig. S6). Both roles could be important for these LAADs to function properly. The further studies are needed to see if other membrane proteins could regulate the INS-membrane interaction and then regulate the enzymatic activity of the bacterial membrane-bound LAADs.

## Methods

### Protein expression and purification

The expression plasmids for FL-*pv*LAAD, ΔN-*pv*LAAD and ΔN-ΔINS-*pv*LAAD were constructed previously^25^. FL-*pv*LAAD and ΔN-*pv*LAAD plasmids containing I345A, L347A, L351A, I352A and F355A mutations were generated using QuikChange XL site-directed mutagenesis kit (Agilent Genomics) following the instruction manual and confirmed by DNA sequencing. The *E. coli* M15 (Qiagen) cells carrying *pv*LAAD plasmids were grown at 37 °C overnight in LB medium supplemented with ampicillin (100 μg/mL). The overnight culture was inoculated (1:100) into fresh LB and shaken at 37 °C until the culture reached an OD600 of about 0.5. Then protein expression was induced with 0.1 mM isopropyl-β-d-thio-galactoside (IPTG) for 16 h at 20 °C. The cells were harvested by centrifugation at 4000 rpm for 30 min at 4 °C and resuspended with binding buffer (500 mM NaCl, 50 mM Tris-HCl, pH 8.0, 20 mM imidazole, 5% glycerol). After sonication, cell lysates were centrifuged at 18000 rpm for 30 min, and the supernatants were loaded onto Ni-NTA columns (Qiagen) pre-equilibrated with the binding buffer. The Ni-NTA columns were washed with 20 column volumes of binding buffer to remove impurity, and then the target proteins were eluted with 30 mL of elution buffer (500 mM NaCl, 50 mM Tris-HCl pH 8.0, 200 mM imidazole, 5% glycerol). The *pv*LAAD proteins were further purified with size-exclusion chromatography (HiLoad 16/60 Superdex 200 pg, GE healthcare). The purified *pv*LAAD proteins were desalted and concentrated to about 30 mg/mL in storage buffer (50 mM NaCl, 5 mM Tris-HCl pH 8.0, 10% glycerol), and stored at −80 °C.

### Liposome preparation

*E. coli* total lipid extracts were purchased from Avanti Polar Lipids, Inc. Firstly, 10 mg of lipids were dissolved in 1 mL of chloroform in a round bottom bottle. The solvent was evaporated under reduced pressure on a rotary evaporator. The sample was further dried under nitrogen gas for 2 hours, which resulted a thin lipid film at the bottom of the bottle. The lipid film was resuspended with 2.5 mL of membrane-binding buffer (50 mM NaCl, 5 mM Tris-HCl, pH 8.0) and shaken at 25 °C, 200 rpm for one hour. Then, the lipid suspension was sonicated (200 W, 6 mm microtip probe) for 15 minutes to produce a translucent liposome solution. The liposome solution was transferred to a new eppendorf tube and stored at 4 °C before use.

### *E. coli* membrane preparation

The *E. coli* membrane was prepared using DH5α cells, which do not express any membrane-bound LAAO/LAAD. 500 mL of DH5α cells were grown overnight at 37 °C and harvested by centrifugation (4,000 rpm, 30 min, 4 °C). Cells were suspended in 20 mL of washing buffer (400 mM NaCl, 50 mM Tris-HCl, pH 8.0) on ice, and disrupted by sonicator (20 min, 200 W). Then cell lysate was centrifuged at 20,000 × g for 30 min to remove insoluble proteins and cell debris. The supernatant was collected and subjected to next centrifugation at 100,000 × g for 1 h at 4 °C. After dumped the supernatant, the membrane pellet was weighted and solved to a concentration of 4 mg/mL with buffer of 50 mM potassium phosphate pH 7.5. In order to remove the proteins integrated in or associated with membrane, the membrane solution was treated with pronase E (0.1 mg/mL) at 37 °C. Then the membrane was re-pelleted by centrifugation, and was washed with washing buffer twice to remove the residual pronase E. The membrane fraction was finally suspended in buffer, and the residual proteins were analyzed with 15% SDS/PAGE.

### Membrane binding assay

Each *pv*LAAD protein (20 μM in final) was incubated with protein-free *E. coli* membrane (4 mg/mL in final) in 400 μL of membrane-binding buffer (50 mM NaCl, 5 mM Tris-HCl, pH 8.0) at 25 °C for 30 min. The membrane was pelleted by centrifugation using a Beckman 90 Ti rotor (30 min, 100,000 × g, 4 °C), and the supernatant containing the unbound proteins were collected into another tube. The membrane pellet was washed in membrane-binding buffer once and re-pelleted by centrifugation. The resulting pellets were resuspended in 200 μL buffer of 50 mM NaCl, 5 mM Tris-HCl, pH 8.0 and 5% SDS. Then, the *pv*LAAD proteins co-pelleted with membrane were analyzed using 15% SDS/PAGE. The supernatants during the membrane binding assays were also concentrated to 200 μL and analyzed by SDS/PAGE.

### Catalytic activity measurement

L-phenylalanine, one of the best substrates for *pv*LAAD, was chosen to evaluate the catalytic activities of different *pv*LAAD truncations and mutants. The transformation of L-phenylalanine by *pv*LAADs was measured by detecting the production of phenylpyruvic acid (PPA), which has an absorption peak at 321 nm. In brief, L-phenylalanine was dissolved to the concentration 25 mM in 500 μL of reaction buffer (50 mM potassium phosphate, pH 7.5) with or without 2 mg/mL of *E. coli* membrane. The *pv*LAADs were added at the final concentration of 10 μM to start the reactions. Reactions were incubated at 25 °C, and at the three time points (1 min, 10 min and 60 min), aliquots of 100 μL of the reactions were transferred into a 96-wells plate containing 50 μL of 3 M NaOH. The absorption at 321 nm was measured using microplate reader (Flex Station 3, Molecular Devices). The reaction without adding *pv*LAAD enzyme was used as the blank control. For quantification, phenylpyruvic acid (Sigma-Aldrich) at different concentrations (100 μM, 500 μM, 1000 μM, 2500 μM, 5000 μM, 10000 μM) were used to calculate the standard curve.

### Kinetic parameter determination

*K*_*m*_ and *V*_*max*_ values of the *pv*LAADs against L-phenylalanine were calculated by measuring the phenylpyruvate production rate at the increasing substrate concentrations (0.78125, 1.5625, 3.125, 6.25, 12.5, 25, 50, and 100 mM) in reaction buffer at 37 °C. The reaction initiated by addition of a final concentration of 5 μM of the *pv*LAADs. Reactions were incubated for 5 min and then stopped by adding 3 M NaOH (final concentration is 1 M). The absorption at 321 nm was measured for the reactions. The kinetic parameters Km and Vmax were calculate using the Lineweaver-Burk plotting^36^, which follows the Eq. (1):

1 $$1/V=({K}_{m}/{V}_{{\max }}\ast 1/[{\rm{S}}])+1/{V}_{{\max }}$$

where *V* is the reaction rate (the amount of PPA produced by 1 mg of the *pv*LAAD protein per min), Vmax is the maximum reaction rate, *K*_*m*_ is the Michaelis constant (mM), and [S] is the concentration of L-phenylalanine (mM). Each reaction group was repeated three times, and the results were expressed as the mean ± standard deviation (n = 3).

### Peroxide detection

Peroxide was determined by a method based on the commercial Amplex® Red Glutamic Acid/Glutamate Oxidase Assay Kit (Invitrogen). The reaction mixture contained 25 mM L-phenylalanine, 10 μM *pv*LAAD protein and with or without 2 mg/mL of *E. coli* membrane. Reactions were incubated at 37 °C for 60 min. Then, working solution (100 μM Amplex® Red reagent, 0.25 U/mL HRP) was added, and the reactions were incubated at 37 °C for 40 min. Then, fluorescence emission at 585 nm (excited at 571 nm) were recorded using a microplate reader (Flex Station 3, Molecular Devices). The reactions without *pv*LAAD protein were used as the blank control. The standard curve of H_2_O_2_ concentrations was generated using H_2_O_2_ samples at different concentrations (5000, 2000, 1000, 500, 250, 125, 62.5 μM).

### Substrate selectivity measurement

10 μL of enzymes (200 μM) were mixed with 90 μL of one of the twenty native L-amino acids (10 mM) with or without *E. coli* membrane (2 mg/mL). Reactions were incubated at 25 °C for 60 min, and then 100 μL trichloroacetic acid (20%) were added to stop the reactions. After that, 40 μL 2,4-dinitrophenylhydrazine (DNP, 20 mM) was added to produce the corresponding 2,4-dinitrophenylhydrazone derivatives. After incubating at 37 °C for 15 min, 800 μL of NaOH (0.8 M) was added to stop the reactions. Additional 5 min incubation was applied at room temperature. Finally, the reactions were transferred into the plastic cuvettes and absorbance at 520 nm were measured. The concentrations of α-keto acid were calculated according to the standard curve generated by different concentrations (25, 100, 250, 500, 1000 and 2000 μM) of phenylpyruvate (Sigma-Aldrich).

### Circular dichroism difference spectroscopy

CD spectra were recorded using a Chirascan with the 0.1 cm cuvettete (Hellma). Spectra for the liposome (2 mg/mL), *pv*LAADs (20 μM) and their mixture were collected in the wavelength range of 200–260 nm using the bandwidth of 1 nm and the resolution of 1 nm. Three parallel spectra for each sample were recorded and averaged. Spectrum for the buffer (50 mM NaCl, 5 mM Tris-HCl, pH 8.0) was collected as baseline and was subtracted during data process. Spectrum of the *pv*LAADs bound with the liposome was calculated by deducting the spectrum of the liposome sample from that of *pv*LAAD-liposome mixture. Data were expressed as mean residue ellipticity: [θ] (degree·cm^2^/dmol). The fractional percentage of the secondary structure was calculated by the Dichro-Web^37^.

### Molecular dynamic simulations

Crystal structure of ΔN-*pv*LAAD (residues 30–471, PDB code 5hxw) was used as the initial model, and prepared using Molecule Operating Environment (MOE, Chemical Computing Group Inc.) package. The protonation states of charged residues were determined by H++ program^38^, and then the model was neutralized by adding Na^+^ or Cl^−^ ions at protein surface using Amber^39^. The model was solvated into a box with a 10 Å buffer distance between the solvent box wall and the nearest solute atoms. The TIP3P model^40^ and Amber99SB force field^41^ were applied for water molecules and the proteins, respectively. The model was firstly minimized, and then gradually heated from 0 to 300 K over a period of 50 ps, followed by another 100 ps of isothermal-isobaric ensemble (NPT) MD simulations to relax the system density to about 1.0 g/cm^3^, with the target temperature of 300 K and the target pressure of 1.0 atm. Afterward, 160 ns of canonical ensemble (NVT) MD simulations with a target temperature of 300 K via employing the periodic boundary condition were performed to produce trajectories. RMSD-based clustering was performed with the program *ptraj* implemented in Amber12.

## Electronic supplementary material

Supplementary Information
