# Supplementary material for: Membrane binding of the insertion sequence of Proteus vulgaris L-amino acid deaminase stabilizes protein structure and increases catalytic activity
Source: Sci Rep. 2017 Oct 20;7:13719. doi: 10.1038/s41598-017-14238-7 (PMC5651824; doi:10.1038/s41598-017-14238-7)
Supplement: Supplementary file 1 — Supplementary Information [file 41598_2017_14238_MOESM1_ESM.pdf]

SUPPLEMENTARY INFORMATION

Membrane binding of the insertion sequence of *Proteus vulgaris* L-amino acid deaminase stabilizes protein structure and increases catalytic activity

Yingchen Ju[1], Zhihong Liu[1], Zizhen Zhang[1], Lijun Duan[1], Qi Liu[1], Qiong Gu[1], Cheng Zhang[2], Jun Xu[1,*], and Huihao Zhou[1,*]

[1]Research Center for Drug Discovery, School of Pharmaceutical Sciences, Sun Yat-sen University, Guangzhou, 510006, China

[2]Department of Pharmacology and Chemical Biology, School of Medicine, University of Pittsburgh, Pittsburgh, 15261, USA

[*]Correspondence and requests for materials should be addressed to H.Z. (zhuihao@mail.sysu.edu.cn) and J.X. (junxu@biochemomes.com)

Supplementary Figures S1-S6

Supplementary Table S1

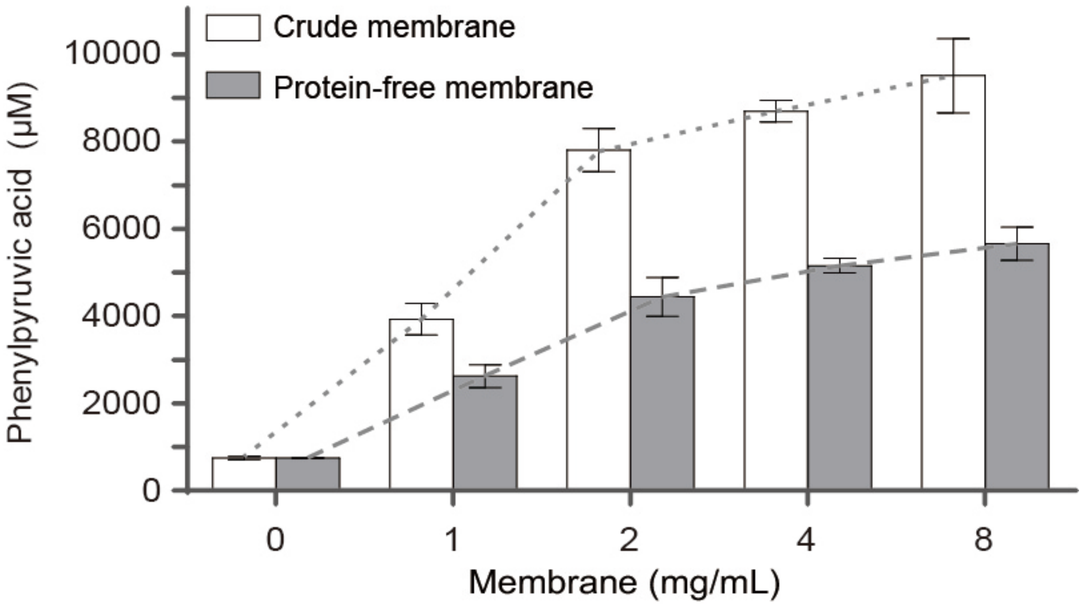

Supplementary Figure S1. The activity of wild-type FL-*pv*LAAD was increased by different amounts of membrane. The activities of wild FL-*pv*LAAD with crude membrane extract (white column) and protein-free membrane (gray cloumn) at a series of concentrations (0, 1, 2, 4, and 8 mg/mL) were mearsured after 60 min. For both the crude membrane and protein-free membrane, the activity increase of *pv*LAAD by membrane grew fast when membrane amount was increased from 0 mg/mL to 2 mg/mL. However, after that, further activity increase by adding excess amount of membrane was limited.

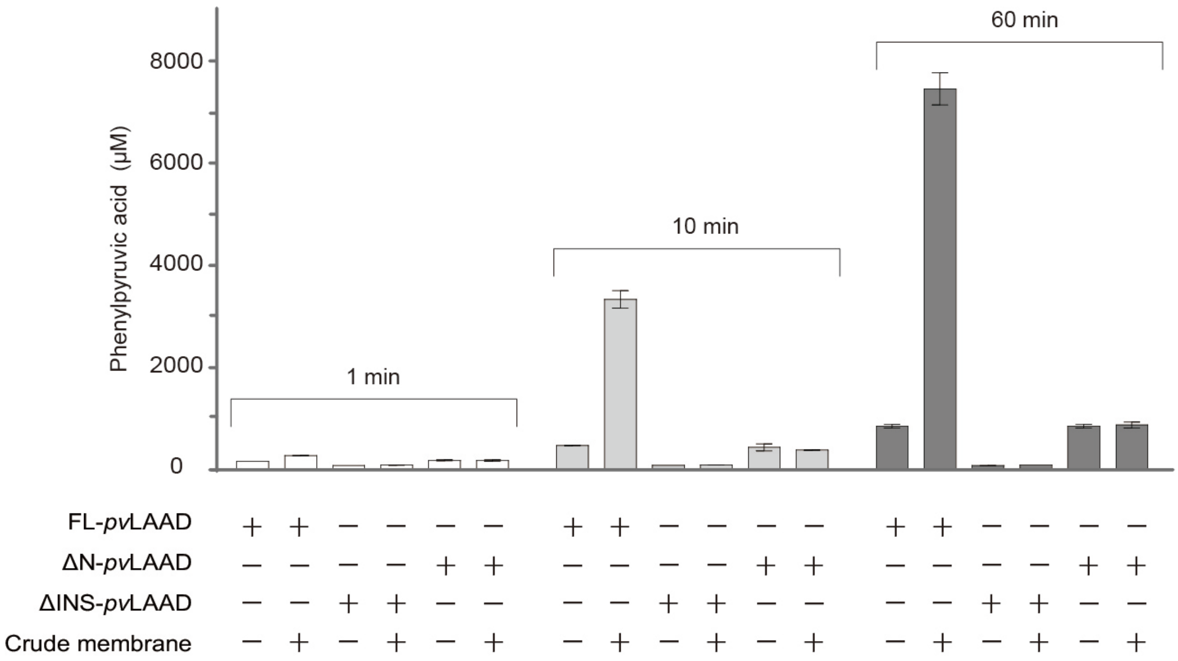

Supplementary Figure S2. The enzymatic activities of the intact and truncated *pv*LAADs with or without membrane presence. The activities were tested by measuring the transformation of L-phenylalaine to phenylpyruvic acids (PPA) in 1, 10 and 60 min. The intact *pv*LAAD (FL-*pv*LAAD) and N-terminus-truncated *pv*LAAD (ΔN-*pv*LAAD) showed similar activity without membrane. Deletion of the INS (ΔINS-*pv*LAAD) made the *pv*LAAD lost almost all of its catalytic activity, indicating that the INS plays a key role in catalysis. The bacterial membrane only enhanced the catalytic activity of FL-*pv*LAAD, but has no effect on ΔN-*pv*LAAD and ΔINS-*pv*LAAD.

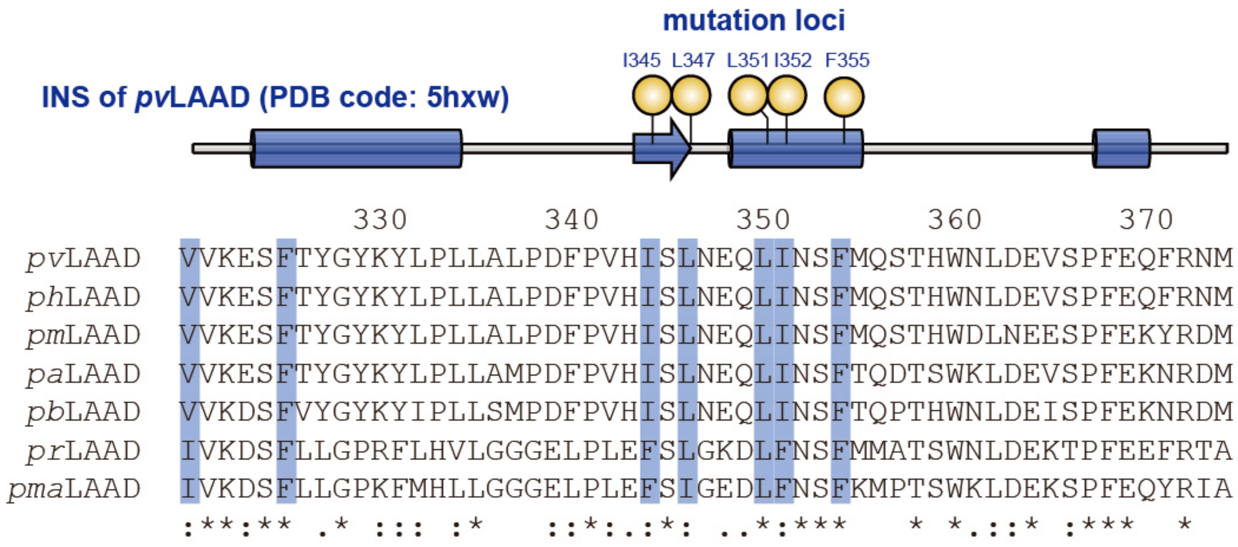

Supplementary Figure S3. The sequence alignments of the INS and mutation sites. The INS sequences of *Proteus vulgaris* L-amino acid deaminase (*pv*LAAD, GeneBank accession number BAA90864), *Proteus hauseri* ZMd44 L-amino acid deaminase (*ph*LAAD, GeneBank accession number EST56802), *Proteus mirabilis* L-amino acid deaminase (*pm*LAAD, GeneBank accession number ACD36582), *Providencia alcalifaciens* Dmel2 L-amino acid deaminase (*pa*LAAD, GeneBank accession number EKT66496), *Providencia burhodogranariea* DSM 19968 L-amino acid deaminase (*Pb*LAAD, GeneBank accession number EKT62719), *Providencia rettgeri* Dmel1 L-amino acid deaminase (*pr*LAAD, GeneBank accession number EKT57949) and *Proteus myxofaciens* L-amino acid deaminase (*pma*LAAD, PDB code 5FJM_A) were aligned using ClustalW program (http://www.genome.jp/tools-bin/clustalw). The secondary structures of the INS were drawn based on the crystal structure of *pv*LAAD (PDB code 5hxw). Seven conserved surface-exposed hydrophobic residues in membrane-bound LAADs were colored blue. The yellow globule represented the five residues which were chosen to mutate in the assays. .

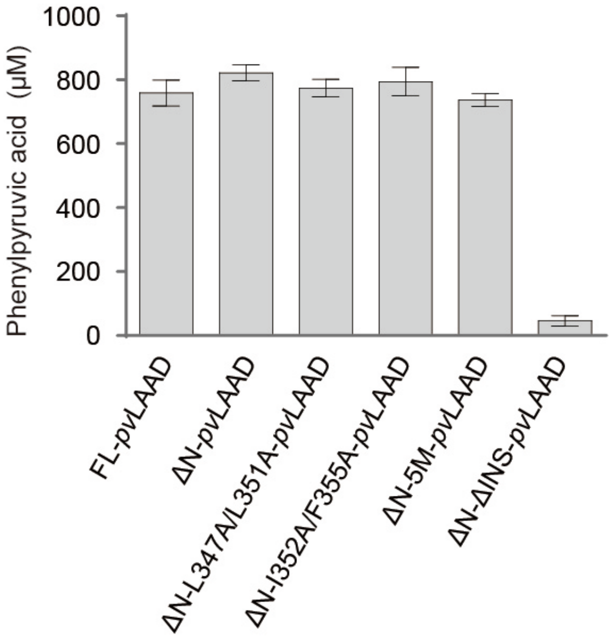

Supplementary Figure S4. Catalytic activities of the wild type and mutated *pv*LAADs in the solution. Similar to the previous results, truncation of the N-terminal transmembrane peptide did not affect the activity of *pv*LAAD, and further deletion of the INS completely blocked its catalytic activity. However, the site mutations in the INS did not affect the enzymatic activity of *pv*LAAD in the solution.

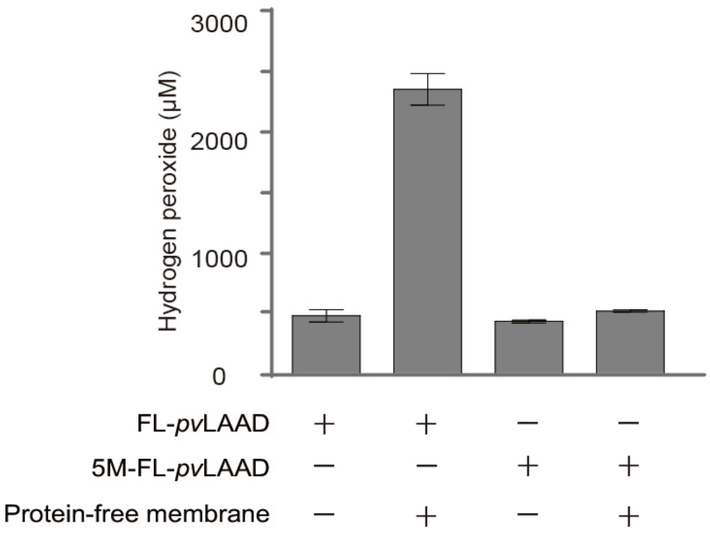

Supplementary Figure S5. The amount of H_2O_2 produced by FL-*pv*LAAD and 5M-FL-*pv*LAAD with or without membrane presence. The H_2O_2 productions of FL-*pv*LAAD and 5M-FL-*pv*LAAD with or without protein-free membrane were measured after 60 min using HRP-coupled assays. For the wild-type FL-*pv*LAAD, the H_2O_2 production was significantly increased by the membrane compared to without membrane (about 4.7 folds). In contrast, for the 5M-FL-*pv*LAAD, the membrane did not show any increase on the H_2O_2 production. This result suggested that the INS-mediated membrane binding affects the activity of *pv*LAAD.

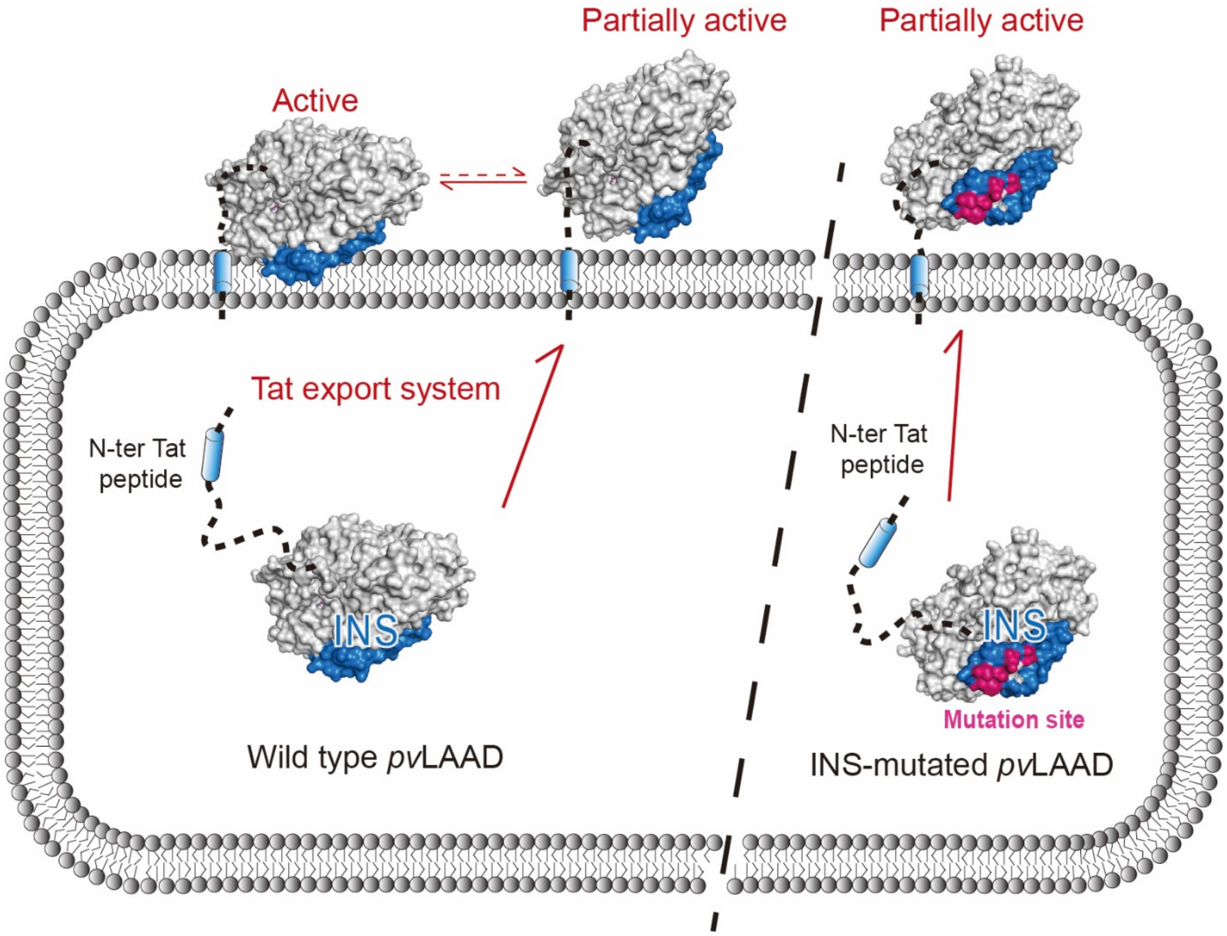

Supplementary Figure S6. The activity regulation of *pv*LAAD in bacteria. The new synthesized *pv*LAAD polypeptides fold in cytoplasm where cofactor FAD exists. The N-terminal Tat peptide will translocate *pv*LAAD across cytomembrane and then anchors it to bacterial surface. When the INS binds to bacterial membrane, its conformation will be stabilized and the *pv*LAAD will be fully activated. Because of the weak binding affinity, it is possible that the INS can dissociate from bacterial membrane when the high enzymatic activity is not required. If the INS-membrane interaction was abolished by mutations in the INS, *pv*LAAD could not be fully activated, and the membrane-bound mutated *pv*LAAD shows similar activity to that in the solution. The INS is shown as the blue surface, the mutated hydrophobic patch is colored magenta, and other parts of *pv*LAAD is colored white.

Supplementary Table S1. Percentage of secondary structure components of *pv*LAADs in the solution or on the liposome.

**Secondary structure components**			**α-Helix**	**β-Sheets**	**Random coil**
**ΔN-*pv*LAAD**	X-Ray(%)		29	22	49
**FL-*pv*LAAD**	X-Ray(%)		30	23	47
	CD spectroscopy (%) (this study)	in buffer	31	24	45
		with liposome	35	27	38
**5M-FL-*pv*LAAD**	CD spectroscopy (%) (this study)	in buffer	29	23	48
		with liposome	30	23	47
